# Delayed sleep–wake phase disorder and its related sleep behaviors in the young generation

**DOI:** 10.3389/fpsyt.2023.1174719

**Published:** 2023-05-19

**Authors:** Kunihiro Futenma, Yoshikazu Takaesu, Yoko Komada, Akiyoshi Shimura, Isa Okajima, Kentaro Matsui, Kosuke Tanioka, Yuichi Inoue

**Affiliations:** ^1^Department of Neuropsychiatry, Graduate School of Medicine, University of the Ryukyus, Okinawa, Japan; ^2^Japan Somnology Center, Neuropsychiatric Research Institute, Tokyo, Japan; ^3^Institute for Liberal Arts, Tokyo Institute of Technology, Tokyo, Japan; ^4^Department of Psychiatry, Tokyo Medical University, Tokyo, Japan; ^5^Department of Psychological Counseling, Faculty of Humanities, Tokyo Kasei University, Tokyo, Japan; ^6^Department of Clinical Laboratory, National Center Hospital, National Center of Neurology and Psychiatry, Tokyo, Japan; ^7^Department of Sleep-Wake Disorders, National Institute of Mental Health, National Center of Neurology and Psychiatry, Tokyo, Japan; ^8^Department of Somnology, Tokyo Medical University, Tokyo, Japan

**Keywords:** delayed sleep–wake phase disorder, DSWPD, circadian rhythm sleep–wake disorder, adolescent and young adult, circadian-entrained DSWPD

## Abstract

Delayed sleep–wake phase disorder (DSWPD) is a sleep disorder in which the habitual sleep–wake timing is delayed, resulting in difficulty in falling asleep and waking up at the desired time. Patients with DSWPD frequently experience fatigue, impaired concentration, sleep deprivation during weekdays, and problems of absenteeism, which may be further complicated by depressive symptoms. DSWPD is typically prevalent during adolescence and young adulthood. Although there are no studies comparing internationally, the prevalence of DSWPD is estimated to be approximately 3% with little racial differences between Caucasians and Asians. The presence of this disorder is associated with various physiological, genetic and psychological as well as behavioral factors. Furthermore, social factors are also involved in the mechanism of DSWPD. Recently, delayed sleep phase and prolonged sleep duration in the young generation have been reported during the period of COVID-19 pandemic-related behavioral restrictions. This phenomenon raises a concern about the risk of a mismatch between their sleep–wake phase and social life that may lead to the development of DSWPD after the removal of these restrictions. Although the typical feature of DSWPD is a delay in circadian rhythms, individuals with DSWPD without having misalignment of objectively measured circadian rhythm markers account for approximately 40% of the cases, wherein the psychological and behavioral characteristics of young people, such as truancy and academic or social troubles, are largely involved in the mechanism of this disorder. Recent studies have shown that DSWPD is frequently comorbid with psychiatric disorders, particularly mood and neurodevelopmental disorders, both of which have a bidirectional association with the pathophysiology of DSWPD. Additionally, patients with DSWPD have a strong tendency toward neuroticism and anxiety, which may result in the aggravation of insomnia symptoms. Therefore, future studies should address the effectiveness of cognitive-behavioral approaches in addition to chronobiological approaches in the treatment of DSWPD.

## 1. Introduction

Circadian rhythm, which runs for approximately 24 h, is present in all animate beings and acts as a regulatory mechanism that promotes optimal adaptation to various biological activities, including not only sleep and wakefulness but also various biological activities such as feeding, reproduction, and social activities (1). Normally, when circadian rhythms are synchronized with the light–dark cycle of the external world, melatonin is secreted as light exposure diminishes during the night and sleep is initiated during the downward phase of the core body temperature (CBT). Among the circadian rhythm markers, dim light melatonin onset (DLMO) and CBT rhythms show a phase relationship with the 24-h cycle. The relationship between the circadian rhythm markers (i.e., DLMO or CBT rhythm) and sleep timing (i.e., sleep onset or offset) is called the “phase angle” of circadian entrainment. Both Earth’s rotation and social activity rhythms run on the 24-h cycle, but the endogenous circadian rhythm in humans may be typically a little longer than 24 h. The length of the intrinsic circadian period is called “tau.” Although the length of tau varies from individual to individual, the mean period of tau is reported to be 24.15 h (standard deviation 0.2 h) (2–4), with the length of tau of women being approximately 6 min shorter than that of men (5). Endogenous circadian rhythms are entrained to follow a 24-h cycle of the external world by various zeitgebers, which are the external factors that serve as cues for entrainment. Among these, light stimulation at a certain time of the day alters the firing rate of neurons in the brain’s suprachiasmatic nucleus (SCN; the command center of the circadian clock), activates the molecular signaling pathway, and alters the transcription of clock genes that determine the phase of the circadian cycle by regulating the rhythm of endogenous melatonin secretion (6). The effect of circadian phase resetting through melatonin and photic stimulation in the SCN follows the phase-response curve (PRC). In the PRC, if light is applied before the minimal point of CBT (CBTmin) appears, the melatonin secretion phase is likely to be delayed, whereas if light is applied after CBTmin, the phase is likely to advance (7). In a normal lifestyle, evening light delays the circadian clock by delaying the sleep onset timing, whereas morning light advances the circadian clock. Light exposure is the most important entrainment factor, but other zeitgebers, such as exercise, mealtime, and social activities also contribute to circadian rhythm entrainment. However, the entrainment ability of these factors is weaker than that of light (8, 9).

Many adolescents and young adults worldwide exhibit a delayed sleep pattern, which can be considered a disorder when it significantly affects important areas of an individual’s functioning. Delayed Sleep–Wake Phase Disorder (DSWPD) (10) is a circadian rhythm disorder in which the delay of sleep phase causes difficulty in falling asleep and waking up at a desired time, resulting in daytime dysfunction. This disorder is associated with multiple factors including specific biological traits, socio-psychological backgrounds, sleep hygiene problems, and comorbid psychiatric diseases. DSWPD is prevalent during adolescence and young adulthood. The establishment of treatment strategies against this disorder is therefore important because the incidence of DSWPD during these critical developmental stages can damage an individual’s future prospects. However, the pathological mechanism of DSWPD as well as many aspects such as the adequate classification, assessment, and treatment strategy of subgroups based on patients’ backgrounds, psychosocial characteristics, and physiological findings remain unclear. Furthermore, recent changes in the light environment surrounding adolescents have increased the likelihood of eveningness chronotype possibly leading to the development of DSWPD (11). A significant number of young people do not meet the diagnostic criteria of DSWPD but have delayed sleep phases (DSP) (12). Social jetlag (SJL) is a new concept that refers to the difference in sleep timing between weekdays and rest days has also been proposed as a concern regarding circadian rhythm related sleep hygiene in young people (13).

The development of DSWPD reflects the multifaceted interaction between social schedules, timing of exposure to light and dark, genetic factors, homeostatic pressure on sleep, and the circadian system. The extent to which a combination of any of these factors is impaired is difficult to identify in a clinical setting. Moreover, approximately 40% (14) of patients with DSWPD have normal timing of melatonin secretion profile (the most important marker of circadian rhythm) even though their sleep–wake schedule is clearly delayed. This subgroup of DSWPD without circadian misalignment is termed circadian-entrained DSWPD and occurs based on the psychological and behavioral backgrounds in addition to biological factors in the young generation (15). Recent studies have also shown that DSWPD is frequently comorbid with some psychiatric disorders, particularly neurodevelopmental and mood disorders, both of which have a bidirectional association with the pathophysiology of DSWPD. In 2015, the American Academy of Sleep Medicine (AASM) published revised guidelines for the treatment of circadian rhythm sleep–wake disorders (CRSWDs), including DSWPD. However, additional treatment methods for circadian-entrained DSWPD and DSWPD complicated by psychiatric disorders should be established.

In this review, we describe the physiological and psycho-behavioral backgrounds of circadian-entrained and non-entrained DSWPD in young people, as well as the relationship between psychiatric disorders and DSWPD. Furthermore, we discuss the current problems and future development of the treatment of this disorder based on the results of psychological and psychiatric assessments.

## 2. Features of delayed sleep–wake phase disorder

### 2.1. Diagnosis of delayed sleep–wake phase disorder

In 1981, Weitzman et al. (16) first proposed the concept of delayed sleep phase syndrome (DSPS) as a chronobiological disorder in a group of patients with chronic difficulty falling asleep and waking, distinguishing it from insomnia and hypersomnia. The concept of DSPS, along with sleep disorders in shift workers, jet lag, and other chronobiological disorders, was included in the first edition of the International Classification of Sleep Disorders (ICSD) (17) as a group of circadian rhythm sleep disorders (CRSD). In the second edition (ICSD-2) (18), DSPS was conceptualized from a syndrome to a single disorder and was referred to as CRSD, delayed sleep phase type (DSPT), or delayed sleep phase disorder (DSPD). However, it is now referred to as DSWPD in ICSD-3 (10). In ICSD-3, the category of CRSD was also renamed circadian rhythm sleep–wake disorder (CRSWD). The ICSD-3 criteria of CRSWD consist of the following three items. First, chronic or recurrent pattern of sleep–wake rhythm disruption primarily because of alteration in the endogenous circadian timing system or misalignment between the endogenous circadian rhythm and the sleep–wake schedule that is desired or required by an individual’s physical environment or social/work schedules. Second, circadian rhythm disruption that leads to insomnia symptoms, excessive sleepiness, or both. Third, sleep and wake disturbances that cause clinically significant distress or impairment in mental, physical, social, occupational, educational, or other important areas of functioning.

The ICSD-3 (10) classifies CRSWD into the following seven categories: (a) DSWPD, (b) advanced sleep–wake phase disorder (ASWPD), (c) irregular sleep–wake rhythm disorder (ISWRD), (d) non-24-h sleep–wake rhythm disorder (N24SWD), (e) shift work disorder (SWD), (f) jet lag disorder (JLD), and (g) circadian sleep–wake disorder not otherwise specified (NOS). DSWPD is the most common sleep–wake disorder, accounting for 83% of CRSWDs in clinical settings (19). In addition to the above three common criteria items for CRSWD, the diagnostic criteria for DSWPD in ICSD-3 requires the fulfillment of the following five criteria. First, a significant delay in the phase of the major sleep episode in relation to the desired or required sleep time and wake-up time, as evidenced by a chronic or recurrent complaint by the patient or caregiver of the inability to fall asleep and difficulty awakening at a desired or required clock time. Second, symptoms are present for at least 3 months. Third, patients exhibit improved sleep quality and duration for their age and maintain a delayed phase of the 24-h sleep–wake pattern when they are allowed to choose their *ad libitum* schedule. Fourth, sleep log and, whenever possible, actigraphy monitoring for at least 7 days (preferably 14 days) demonstrates a delay in the timing of the habitual sleep period. Fifth, sleep disturbance is neither satisfactorily explained by another current sleep disorder nor by any medical, neurological, or mental disorder; medication use; or substance use disorder.

### 2.2. Symptomatic characteristics of DSWPD

The sleep duration of patients with DSWPD is mostly well maintained during their free days, although the sleep phases are apparently delayed. However, on weekdays, they experience difficulty falling asleep and waking up at socially desirable times, such as school or work timings, which interferes with their daily lives. Patients with DSWPD frequently experience daytime sleepiness, fatigue, headache, anorexia, and depression. DSWPD with a regressed rhythm of melatonin secretion, as well as other hormones such as cortisol, may also present with decreased blood pressure during the daytime. This is particularly observed in the morning hours and is often manifested as orthostatic dysregulation (20). Patients with DSWPD with severely delayed circadian rhythms may also exhibit serious sleep inertia when attempting to wake them up at socially desirable times.

Many studies have reported a certain relationship between DSWPD and psychological problems or decreased social functioning, although some negative observations have also been reported (21). Cross-sectional studies have shown higher depression and anxiety scores in patients with DSWPD (14, 22–25), as well as lower health-related quality of life (25) and more frequent self-harm and suicidal ideation (26). Individuals with DSWPD may be unable to adjust to school life or employment (12, 27) when their symptoms are severe. In contrast, the removal of the constraints of attendance at school or work, the most important social zeitgebers, may worsen the symptom severity of the disorder (28). DSWPD has been suggested to be associated with lower grades in students (23, 29) and frequent absenteeism, as well as lower productivity and presenteeism in the working generation (25, 30). Socially, patients with DSWPD tend not to enjoy leisure time and are unable to fulfill their household responsibilities (30). Substance use issues, such as smoking, alcohol, caffeine, and cannabis, may also arise (12, 21, 23, 31). The above associations observed in patients with DSWPD are sometimes observed in individuals with the evening chronotype (32), which suggests that DSWPD is possibly an extreme phenotype of the eveningness chronotype.

### 2.3. Assessment of DSWPD

The diagnosis of DSWPD requires a thorough investigation of the medical, mental, or sleep disorders that may cause sleep–wake cycle alterations, insomnia, or excessive daytime sleepiness. Social maladjustment, family dysfunction, school avoidance, and comorbid mood disorders should also be investigated in adolescents and young adults.

A sleep diary (sleep log) monitored for at least 7 days (ideally longer) is mandatory as an indicator for the diagnosis of DSWPD in ICSD-3. Actigraphy, which uses a non-invasive wrist-worn accelerometer, can capture rest-activity rhythms from which the timing and regularity of sleep can be estimated. In the ICSD-3 diagnostic criteria, an actigraphic recording is not necessary for diagnosis (listed as “whenever possible”). However, because sleep diaries may cause misunderstanding and recall bias, supportive actigraphic recording is desirable for objectively measuring an individual’s sleep–wake schedule. In ICSD-3, actigraphic recording is set as a mandatory item only for the diagnosis of N24SWD. Nevertheless, we believe that actigraphic recordings along with a sleep diary for a minimum evaluation period of 14 days are necessary for the accurate diagnosis of DSWPD. Although the information provided by actigraphy is inherently limited with respect to the assessment of the underlying chronobiological complexity associated with CRSWDs (11), previous studies (as shown below) showed that actigraphy can reflect the status of the melatonin secretion profile of patients with DSWPD (33). Alternatively, low burden and relatively inexpensive consumer-grade wearable and mobile technologies are now attracting interest as devices for measuring the conventional biomarkers of sleep (34). However, further studies are necessary to validate whether these devices can serve as a useful assessment tool for an individual’s sleep–wake schedule.

The CBT and melatonin secretory rhythms, which are generated by the SCN in parallel with the circadian sleep propensity rhythm, are well-established indicators of circadian rhythms and often used in clinical studies of DSWPD (35, 36). However, they are difficult to measure in general clinical practice. Previously, CBT assessment was a highly invasive approach that used a rectal probe. In recent years, a simple non-invasive tool that can be attached to the surface of the chest or other body parts has been developed to assess CBT (37, 38). Melatonin secretion can be measured through serial salivary assays, serum assays, or the measurement of urinary 6-sulphatoxymelatonin. Among these, DLMO, which is measured in light less than 10 lx, enables the estimation of the circadian phase of melatonin secretion (35, 39). However, since CBT and melatonin secretion can be obscured by masking effects (e.g., light suppresses melatonin secretion and activity and sleep modifies the CBT rhythm), these measurements are highly recommended to be performed in highly controlled conditions to minimize contamination by the masking effects.

Self-administered chronotype questionnaires have also been commonly used to assess patients highly suspected of having DSWPD. Among these, the morningness-eveningness questionnaire (MEQ) (40) and Munich chronotype questionnaire (MCTQ) (41) have been accepted as reliable chronotype measures. The MEQ can evaluate an individual’s circadian preference, whereas the MCTQ is advantageous in that it can evaluate an individuals’ sleep-midpoint and SJL, both of which are important indicators of circadian rhythm (42). As described later, DSWPD is commonly observed inpsychiatric disorders, particularly bipolar disorder, suggesting the close relation of pathophysiology between bipolar disorder and CRSWDs (43, 44). The Biological Rhythms Interview of Assessment in Neuropsychiatry (BRIAN) was first developed to measure circadian rhythm dysfunction in patients with bipolar disorder (45). However, our recent studies have shown that BRIAN can be effectively used also for the screening and severity assessment of DSWPD without comorbid psychiatric disorders (25, 46).

Although nocturnal polysomnography is not necessary to establish the diagnosis of DSWPD, it should be performed when the existence of other sleep disorders that may be responsible for subjective insomnia and sleep inertia in the morning is suspected. When performed during conventional sleep laboratory hours, the polysomnographic findings of individuals with DSWPD tend to show prolonged latency for sleep onset and normal or relatively long total sleep time, which are consistent with their sleep logs or actigraphic findings (47, 48).

### 2.4. Epidemiology of DSWPD

DSWPD has a prevalence of 0.17–1.51% in the general population (49, 50), which is reported lower than that of DSP (12). In comparison, a survey of 10,220 adolescents aged 16–18 years in Norway found a relatively higher rate of 3.3% (27), while a more recent Norwegian survey of 50,054 students aged 18–35 years also showed a prevalence of 3.3% (26). These results suggest that DSWPD is possibly more prevalent in the younger generation than in the older generations. Similarly, a recent large Japanese survey estimated that 4.3% of the youth (15–30 years) is at risk for DSWPD (25). The higher prevalence of DSWPD in adolescents and young adults may reflect a preference for a “night owl” lifestyle and biological change in this generation (3, 51).

As aforementioned, studies on the sex differences in the prevalence of DSWPD have been inconclusive. The results of previous epidemiological studies demonstrated a higher prevalence in males (12, 26), females (25, 27), or no sex differences (49, 50, 52). Although the effect of a delayed sleep–wake schedule on the development of depression may be greater in women because of their intrinsically earlier circadian rhythm (53), sex differences in the effect of the disorder on daytime functioning has not been clarified.

No international comparative studies on racial or regional differences in the prevalence of DSWPD have been conducted to date. However, in Germany, people living in the western region of the country have later chronotypes than those living in the eastern region (42). The relatively later sunrise in the western part of the country (although both regions share the same time zone) was speculated to contribute to this difference (54). Table 1 shows a list of the major epidemiological studies on DSWPD over the last decade.

**Table 1:** Prevalence of DSWPD-related disorders by region/country as reported over the past decade.

Region/country (author, year)	Prevalence/sex difference	Study design (*n*/age in years)	Findings
**Europe**			
Norway (Sivertsen, 2021)	3.3% (DSWPD)/male, 4.7% > female, 2.7%**	Cross-sectional study (50,054/18–35)	Single status, financial difficulties, parental divorce, obesity, and physical inactivity were associated with DSWPD.
Norway (Hysing, 2018)	3.9% (DSP)/n.s.	Longitudinal study (2,200/16–19)	Sleep duration of < 9 h/night at the age of 11–13 years was associated with DSP at 16–19 years.
Sweden (Danielsson, 2016)	4.0% (DSPD)/n.s. 4.6% (DSP)/male, 7.3% > female, 2.4%*	Cross-sectional study (10,000/16–26)	DSPD was associated with non-attendance of educational activities or work and elevated levels of anxiety.
Norway (Sivertsen, 2013)	3.3% (DSPS)/female, 3.7% > male, 2.7%*	Cross-sectional study (9,338/16–18)	DSPS was associated with non-attendance at school, with half of the adolescents with DSPS also meeting the criteria for insomnia.
Norway (Saxvig, 2012)	8.4% (DSP)/n.s.	Cross-sectional study (1,285/16–19 years)	DSP was associated with lower average school grades, smoking, alcohol usage, and elevated anxiety and depression scores.
**Asia**			
Japan (Tomishima, 2022)	4.3% (at risk of DSWPD)/ female, 4.9% > male, 2.5%**	Cross-sectional study (7,810/15–30)	Long-term LCD viewing at night and relatively loose social constraints were associated with the presence of DSWPD.
**Oceania**			
New Zealand (Paine, 2014)	1.51% (DSPD)/n.s.	Cross-sectional study (4,386/20–59)	DSPD prevalence was higher in deprived areas and decreased with age. This disorder was associated with the presence of night work.
Australia (Lovato, 2013)	1.1% (DSPD)/n.s.	Cross-sectional study (374/13–18)	Patients with DSPD showed relatively greater alcohol and caffeine consumption, lesser sports participation, and more time spent on extracurricular activities.

DSP, delayed sleep phase; DSPS, delayed sleep phase syndrome; DSPD, delayed sleep phase disorder; DSWPD, delayed sleep–wake phase disorder; LCD, liquid crystal display; n.s., not significant. *p < 0.05, **p < 0.001.

## 3. Pathophysiology of DSWPD

### 3.1. Biological factors of DSWPD

Although the pathogenesis of DSWPD is heterogeneous and complicated by various factors, one of the most important features of typical DSWPD is the delayed circadian rhythm, which is assessed using DLMO or CBT measurements. Many studies have shown a circadian rhythm delay in DLMO in patients with DSWPD (55, 56).

As mentioned in the introduction, light stimulation is the most important zeitgeber; however, individual sensitivity to light varies 50-fold on a logarithmic scale (57). Studies have also shown that light exposure at the same timing and intensity may have different effects on the entrainment phase between individuals (58, 59). In DSWPD, photosensitivity seems to be weak (or the time width of the phase advance is narrow) during the phase advance portion of the circadian PRC to light stimuli (60). Additionally, photosensitivity at night is higher in patients with DSWPD than in normal sleepers, which may contribute to the delay in circadian rhythm (61).

Individual differences in the intrinsic circadian cycle are another cause of DSWPD. The length of tau varies among individuals, and individuals with longer tau are entrained at a later phase than those with shorter tau (62, 63). Furthermore, the circadian cycle of both melatonin secretion and CBT rhythms is longer in patients with DSWPD than in controls, with tau length being associated with the likelihood of developing DSWPD (63, 64). The PERIOD2 (*PER2*) gene encodes a core molecule in the circadian clock and plays an important role in the generation and maintenance of diurnal rhythms. Minor allele carriers of the *PER2* variant have significantly longer circadian cycles than non-carriers, as demonstrated by CBT or plasma melatonin profile (65).

Problems with sleep inertia and sleep architecture may also be related to the pathophysiology of DSWPD. Previously, sleep architecture and sleep duration in patients with DSWPD were considered normal (66). However, several studies have reported prolonged sleep duration in patients with DSWPD (36, 67–69). Moreover, patients with DSWPD have a low amount of slow-wave sleep during the first half of sleep, corresponding to a delay in the timing of CBTmin (69). Patients with DSWPD also have a higher arousal threshold during REM sleep (47) and a prolonged interval between CBTmin and arousal (36, 67, 70). These factors may be related to the difficulty in waking up at a desirable time in the morning, possibly resulting in the decreased light exposure during the phase-advance portion of the PRC.

Chronotype change with age may also be involved in the development of DSWPD. In this regard, some researchers have suggested the role of age and sex differences in the development of DSWPD based on sex hormone changes (71, 72). Gonadal steroid receptors are expressed at most sites that receive direct inputs from the SCN. At each stage of the circadian system, brain nuclei bear estrogen receptors, androgen receptors, or both. From adolescence to young adulthood, the activational effects of sex hormones on the circadian timing system are associated with the phase delay of the circadian rhythm (71). Although the prevalence of DSWPD declines after middle age, a large survey of chronotypes in Brazil (*n* = 14,650) (73) showed that women were on an average more morning-oriented than men up to the age of 30 years. However, from age 30 to 45 years, the sex difference in the chronotype disappeared, and women tended to be more evening-oriented than men aged 45 years and older. The age-related plastic changes associated with sex differences in chronotypes remain largely unknown, however, changes in the sex hormone status may be partially related to the differences in the circadian phases among the respective generations.

Patients with DSWPD have been demonstrated to have a larger phase angle between sleep timing and circadian rhythm markers (67, 69, 74). Polymorphisms or mutations in clock genes may contribute to this expansion of the phase angle (75). Furthermore, DSWPD is frequently associated with difficulty in initiating sleep (27), which may be related to the expansion of the phase angle in DSWPD. Additionally, a sleep homeostatic problem leading to difficulty in increasing sleep pressure has been reported (74).

DSWPD and N24SWD often occur alternately in the same patient, suggesting pathological continuity between the two disorders. A large proportion of individuals with DSWPD as well as those with N24SWD exhibit longer periods of melatonin and temperature rhythms, with longer circadian tau appearing to be the common basis for these disorders (63). N24SWD can occur even in patients without visual impairment and could be an extreme form of DSWPD. On the other hand, DSWPD has also been suggested as the prodromal manifestation of N24SWD without visual impairment (76). In addition to longer intrinsic tau, other potential etiologies shared by DSWPD and N24SWD include altered light sensitivity (77) and homeostatic issues (difficulty in increasing sleep pressure) (63).

### 3.2. Behavioral and social factors in DSWPD

DSWPD appears to be strongly related to youth-specific behavioral factors. Our previous epidemiological study showed that behavioral patterns particular to youth, such as long-term liquid crystal display (LCD) viewing at night, were associated with the presence of DSWPD (25). Considering this, prolonged exposure to LCD screen-based devices, such as TVs, PCs, and smartphones, from evening to bedtime may be associated with the development or worsening of DSWPD. Similarly, the duration of monitor viewing time was also demonstrated to be adversely associated with sleep health, primarily via delayed bedtime and reduced sleep duration among school-aged youth (78). The scarcity of physical exercise habits (21, 25, 26) and the presence of night work (50) and extracurricular activities (21) were also associated with the risk of developing DSWPD. Another longitudinal study (52) showed that sleeping less than 9 h/night at the age of 11–13 years was associated with DSP at 16–19 years. In addition, one study reported smoking and drinking habits as factors associated with the presence of DSWPD (23).

DSWPD is a disorder that can develop on a psychosocial basis. In support of this, our recent study showed that being at risk for DSWPD had a greater association in students than in young adult workers of the same age group (25), suggesting that less social constraints could be associated with the presence of DSWPD in this generation. Several studies have also shown that absenteeism at school or work was associated with DSWPD (12, 27). Thus, the teenagers’ sleep–wake rhythm may be delayed because of their non-attendance at school, which was related to their maladjustment to school or relationship problems. Furthermore, DSWPD in students has been suggested to be associated with financial deprivation, parental divorce (26), and depression (31). However, many of these reports were cross-sectional studies, making the causal relationship unclear. Nevertheless, DSWPD and psychosocial issues may have a bidirectional relationship.

### 3.3. Psychological characteristics of DSWPD

Many researchers have suggested that chronotypes and DSWPD occurrence are associated with specific personality traits. To date, the “Big Five” model, which proposes that personality can be grouped across five broad personality traits that include neuroticism (i.e., emotional instability and moodiness), extroversion (i.e., excitability and sociability), conscientiousness (i.e., thoughtfulness and goal-directed behaviors), agreeableness (i.e., altruism and kindness), and openness (i.e., imagination and insight), has been widely used as the personality trait model in studies on sleep hygiene and chronotypes (79). A meta-analysis of the studies published before the end of January 2009 found that conscientiousness was mostly related to morningness. Moreover, agreeableness was also related to morningness, albeit to a lesser degree (80). In contrast, studies conducted on college students after 2010 reported that high extroversion was associated with eveningness and high conscientiousness, openness, and low neuroticism was related to morningness tendency (81, 82). However, these were cross-sectional studies, and a longitudinal study showed that only low neuroticism predicted morningness 1 year later (83). Another study found that patients with DSWPD had higher neuroticism, lower extroversion, and lower conscientiousness than a healthy control group (84, 85). Taking these findings into consideration, levels of conscientiousness and neuroticism could be associated with the variation in the morningness-eveningness chronotype. Apart from these two personality traits, low extroversion could contribute to DSWPD development. Although few studies have directly examined the relationship between personality traits and sleep-related behavior, low conscientiousness and high neuroticism may become strong predictors of poor sleep hygiene (81) Taking these findings together, certain personality traits may contribute to the development and maintenance of sleep problems including DSWPD through indirect influences on behavioral aspects.

Individuals with DSWPD commonly report difficulty initiating sleep, and 89% of adolescents with DSWPD experience “racing thoughts” in bed (86). Moreover, it has been suggested that individuals with DSWPD may exhibit cognitive pre-sleep arousal (e.g., worry and rehearsal today and planning tomorrow), dysfunctional beliefs about sleep (e.g., “I know that it will not work and then, I sort of just give up”), and safety behaviors (e.g., use of music, television, and computer games as a sleep aid), similar to patients with chronic insomnia (87). Furthermore, an overlap between DSWPD and insomnia has been reported, wherein more than half of the adolescents with DSWPD also met the criteria for insomnia (27). However, whether these cognitive-behavioral characteristics are specific to patients with DSWPD remains unclear.

### 3.4. Neuropsychiatric disorders in DSWPD

#### 3.4.1. Neuropsychiatric disorders and DSWPD

DSWPD is frequently observed in neuropsychiatric disorders, such as major depressive (88); bipolar (89); obsessive–compulsive (90); neurodevelopmental disorders, including attention-deficit hyperactivity disorder (ADHD) and autism spectrum disorder (ASD) (91); and schizophrenia (92). Previous studies have suggested that the presence of DSWPD could result in an increased risk of the occurrence of neuropsychiatric disorders, worsened depressive symptoms, increased relapse risk of mood episodes, and deterioration in social and occupational functioning in the affected individuals. Therefore, accurate diagnosis of DSWPD, definitive biomarkers that can identify the association between DSWPD and psychiatric disorders, and the establishment of a treatment strategy for DSWPD in patients with neuropsychiatric diseases are required. Table 2 shows a list of the major studies investigating the relationship between DSWPD and neuropsychiatric disorders.

**Table 2:** Relationship between psychiatric disorders and circadian rhythm dysfunctions, particularly delayed sleep phase.

Psychiatric disorders (author, year)	Study design participants	Findings
**Mood disorders**		
BD (Takaesu, 2016)	Cross-sectional study 104 euthymic outpatients with BD	Thirty-five participants with BD (32.4%) met the criteria for CRSWD. The presence of CRSWD was associated with a younger onset age of BD and a family history of suicide.
BD (Takaesu, 2018)	Prospective 48-week study 104 euthymic outpatients with BD	Circadian rhythm dysfunction might be a trait marker of BD and risk factor for the relapse of mood episodes.
BD (Steinan, 2016)	Cross-sectional study 404 adults with BD	Younger age, higher BMI, and impairment of energy as well as activity were associated with DSP in adults with BD.
BD (Harvey, 2008)	Review article	Sleep disturbance and circadian dysregulation were critical pathophysiological elements in BD.
BD (Gottlieb, 2019)	Systematic review	Chronobiological treatment focusing on circadian rhythm dysfunction was recommended in the treatment guidelines for BD.
MDD (Robillard, 2018)	Case–control study 34 young adults with MDD and 15 controls	Delayed and disorganized circadian rhythms might be linked to worse psychiatric profiles in young people with depressive disorders.
MDD and BD (Takaesu, 2017)	Case–control study 104 patients with BD and 73 with MDD	The rate of CRSWD in patients with BD was significantly higher than in those with MDD (33.7% vs. 9.6%; *p* < 0.001).
**Neurodevelopmental disorders**		
ADHD (Spera, 2020)	Cross-sectional study 102 adults with ADHD	Thirty-four participants met the criteria for DSWPD, which was associated with young age, cannabis use, cyclothymic temperament traits, and severe global impairment in ADHD.
ADHD (Lunsford-Avery, 2018)	Editorial perspective	Delayed sleep–wake phase could play an important role in the development of late-onset ADHD.
ASD (Carmassi, 2019)	Systematic review	A bidirectional relationship was suggested between circadian sleep dysfunction and ASD.
ASD (Baker, 2017)	Case–control study 36 adults with ASD and normal controls	DSWPD was common in adults with ASD. Employment status, comorbid anxiety, and depression appeared to influence the sleep patterns of the participants with ASD.
**Other psychiatric disorders**		
Schizophrenia (Matsui, 2021)	Cross-sectional study 105 patients with schizophrenia	A total of 18.1% of the patients with schizophrenia had CRSWD. The CRSWD group showed more severe psychiatric symptoms (anxiety) than the non-CRSWD group.
OCD (Nota, 2015)	Review article	Individuals with OCD had shorter sleep duration and higher prevalence of DSPD than controls.

BD, bipolar disorder; MDD, major depressive disorder; CRSWD, circadian rhythm sleep–wake phase disorder; DSP, delayed sleep phase; DSWPD, delayed sleep–wake phase disorder; ADHD, attention-deficit hyperactivity disorder; ASD, autism spectrum disorder; BMI, body mass index; OCD, obsessive–compulsive disorder.

#### 3.4.2. Mood disorders and DSWPD

Many studies have reported a relationship between mood disorders and circadian rhythm dysfunction, including DSWPD. Although the causal relationship between depressive symptoms and delayed sleep–wake phase is unclear, depressive symptoms in individuals with DSWPD have been frequently reported. Abe et al. (22) reported that 46% of patients with DSWPD had moderate-to-severe depressive symptoms, as evaluated using the Zung Self-Rating Depression Scale. In contrast, DSWPD was reported in 9.6% of patients with major depressive disorder (93). Interestingly, another study suggested that DSWPD with delayed DLMO was associated with more severe depressive symptoms than DSWPD without delayed DLMO (14), which implies a pathophysiological relationship between depressive symptoms and circadian rhythm dysfunction.

Circadian rhythm dysfunction may be more prominent in bipolar disorder than in major depressive disorder (93, 94). Many studies have suggested a strong pathophysiological relationship between bipolar disorder and circadian rhythm dysfunction (95, 96), and this dysfunction has been suggested to act both as a trait marker of bipolar disorder and a risk factor for the relapse of mood episodes (43). Based on these results, chronobiological treatment focusing on circadian rhythm dysfunction in bipolar disorder has been recommended in the treatment guidelines for bipolar disorder (97). In particular, bright light therapy has been indicated for depressive symptoms, whereas dark therapy has been suggested for manic symptoms in patients with bipolar disorder (97).

#### 3.4.3. Neurodevelopmental disorders and DSWPD

Recent studies have suggested a significant relationship between circadian rhythm dysfunction and neurodevelopmental disorders, such as ADHD (98) and ASD (99). A study on ASD indicated that a higher proportion of adult patients with ASD met the criteria for CRSWD than adult controls. Moreover, DSWPD was found to be particularly common in individuals with ASD (100). Similarly, a cross-sectional study reported that 34 of 102 adult patients with ADHD met the criteria for DSWPD (91). Considering these findings, delayed sleep–wake phase has been hypothesized to play an important role in the development of ADHD symptoms in late adolescence and young adulthood (101), although no clear evidence supporting this hypothesis has been reported. Therefore, longitudinal studies evaluating the causal relationship between neurodevelopmental disorders and DSWPD are required to understand the pathophysiological relationship between these disorders.

### 3.5. Phenotypes of DSWPD

**Figure 1:**
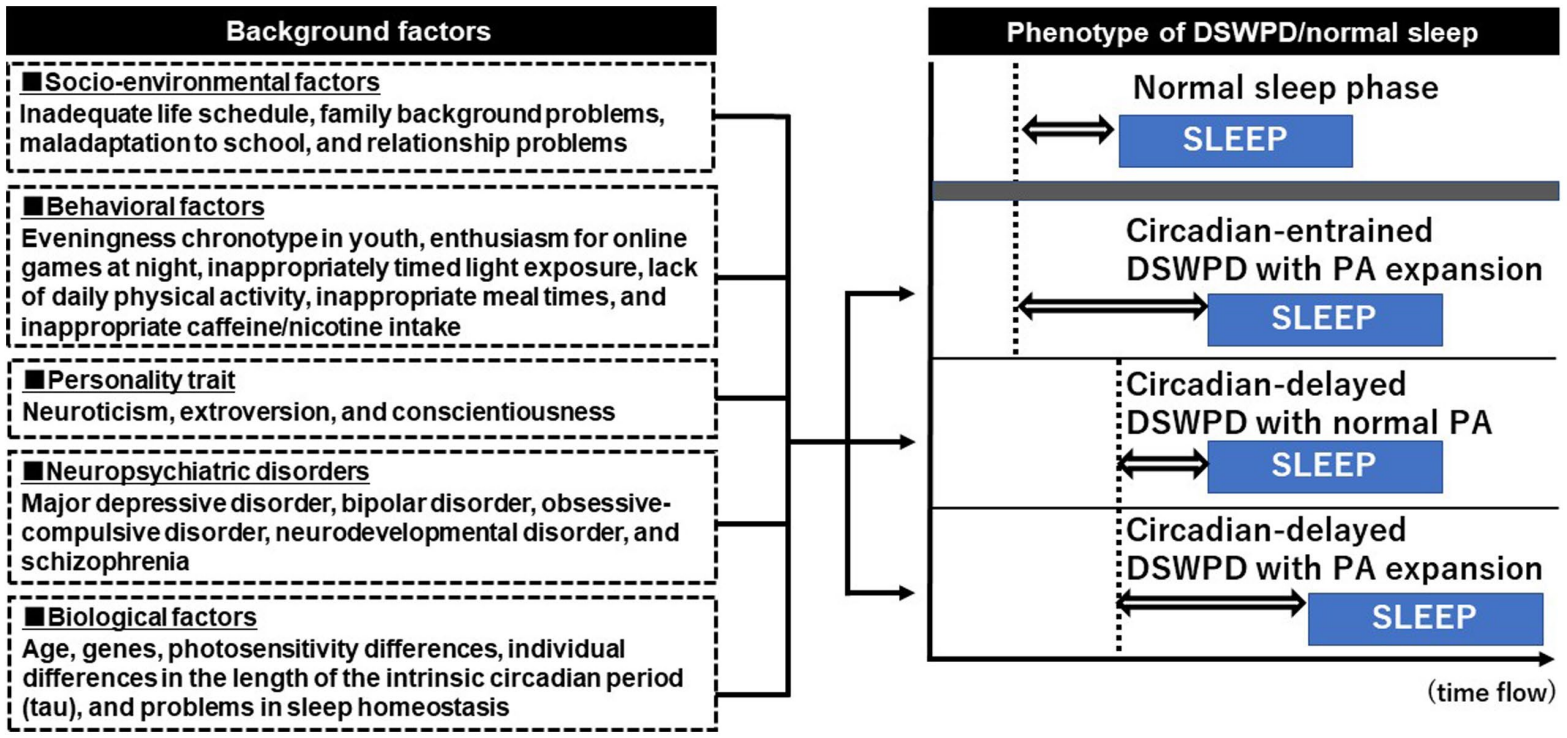
Phenotype of delayed sleep–wake phase disorder and its background factors. The dotted vertical line (---) indicates dim light melatonin onset (DLMO) and the blue box shows the sleep phase. The double-headed arrow (⇔) shows the phase angle (PA) between the DLMO and sleep period onset. The PA between the DLMO and sleep period onset is approximately 2 h in a normal sleeper. Delayed sleep–wake phase disorder (DSWPD) exhibits multiple phenotypes owing to the various combinations of background factors. Approximately 40% of DSWPD cases are reported as circadian-entrained DSWPD, in which the timing of DLMO is normal but the PA is enlarged because of night-oriented behavioral or psychological characteristics. Circadian-delayed DSWPD with PA expansion shows the most severely delayed sleep phase, and this phenotype may have pathological continuity with non-24-h sleep–wake rhythm disorder.

DSWPD is diverse in not only its pathogenesis but also in its phenotypes. DSWPD phenotypes differ depending on a combination of various background factors. Delayed circadian rhythm is the most prominent feature of DSWPD; however, a recent study identified a group of patients with DSWPD having normal circadian entrainment (48, 102). As previously mentioned, 43% of DSWPD cases are circadian-entrained DSWPD (14). Circadian-entrained DSWPD often develops primarily based on problems of behavioral factors (103). “Conditioned insomnia” and “aversion to trying to sleep early” are considered as causes of delayed bedtime in circadian-entrained DSWPD (15). Patients with circadian-entrained DSWPD may be associated with negative experiences with going to bed early or have personality traits (e.g., perfectionism) that interfere with bedtime (e.g., staying up late to complete tasks) (104). In contrast, DSWPD with longer tau and an enlarged phase angle shows the most severe delay of sleep–wake phase, and this phenotype is suggested to have pathological continuity with N24SWD (36). The background factors and phenotypes of DSWPD are shown in Figure 1.

## 4. Recent changes in lifestyle and new concerns of DSWPD in the young generation

### 4.1. Social and environmental changes that may exacerbate eveningness chronotype in the young generation

Compared to the natural light/dark cycle during outdoor camping, the nocturnal light exposure of modern lifestyles are associated with sleep phase delay (105). With this regard, a Finnish study found a decline in sleep duration and an increase in eveningness among the adult population even in the first decade (2007–2017) of the 21^st^ century (106). The effect of light exposure on circadian rhythm differs depending on an individual’s age. Because the lenses in adolescents are relatively more transparent than those of older adults (107), melatonin secretion in adolescents is suppressed even with a relatively small amount of nighttime light exposure that would not affect adults (108). Therefore, nighttime light exposure may become an important risk factor for the development of the eveningness chronotype in this generation (109). Recent changes in the light environment surrounding adolescents have increased the risk of them developing the eveningness chronotype (110). In particular, one study found that the long-term use of smartphones in bed could be a significant risk factor for the eveningness chronotype because using it while lying down and observing the screen at very close range exposes one’s eyes to bright light that exceeds 100 lx (111).

A recent cohort study has shown that the number of individuals who meet the diagnostic criteria of DSWPD and the number of people who only have DSP without any sleep complaints for at least 3 months is roughly equal among those who have a sleep–wake schedule delay (12). Undoubtedly, the pathological significance is higher for individuals with DSWPD; however, DSP in the young generation reportedly possesses identifiable psychological risk indicators (52). In the case of DSWPD, no sex difference exists in its prevalence, whereas elevated anxiety levels and the presence of absenteeism (absence from school or work) have been demonstrated (12). In contrast, DSP is more common in men and is associated with a lack of educational activity or work, the presence of shift work, the use of nicotine and alcohol, and less rumination score. Therefore, individuals with DSP could possibly include a substantial number of those in whom the sleep–wake phase is intentionally delayed by personal preference or lifestyle rather than by biological reasons. The ICSD-3 has subtyped DSWPD with poor motivation for treatment as motivated delayed sleep–wake phase disorder (MDSWPD), which is considered more common in adolescents and young adults with comorbid psychiatric disorders, such as developmental and anxiety disorders (10). Considering that MDSWPD is a state of poor internal motivation to regain a normal social life, many MDSWPD cases could be included in the DSP category. As described later, the chronobiological approach is the mainstay treatment for patients with DSWPD; however, behavioral approach would be a better choice for correcting DSP.

In the youth with eveningness chronotype, SJL is commonly observed along with DSP (13). SJL can be easily measured with the result of the aforementioned MCTQ (42). Individuals with larger SJL are more likely to report excessive daytime sleepiness (112, 113) and daytime dysfunction because of the internal desynchronization caused by circadian phase delay (13, 114), including low cognitive function (115), poor academic performance (116), depression (117), and substance use (118). In order to test the hypothesis that SJL can easily develop during adolescence and young adulthood (119), we previously conducted a cross-sectional survey in a large Japanese population. The result showed that the younger the age, the greater the SJL, with 61% of those in their 20s versus 53% of those in their 30s showing a SJL of >1 h (120). SJL is associated with the delay in circadian rhythms (13), and sometimes exacerbates the problem of falling asleep when resuming weekdays during the following week (121). However, whether SJL is a precursor of DSWPD remains unclear. Moreover, SJL is a relatively new concept and literature on its physiological characteristics and natural course is scarce. Therefore, further research from multiple perspectives is required to delineate the relationship between SJL and DSWPD.

### 4.2. COVID-19 pandemic and sleep behavior in adolescents and young adults

From 2020 to 2022, the novel coronavirus disease (COVID-19) has spread globally. During this period, sleep disturbances were observed in up to two-fifths of the general population and up to three-fourths of the patients with COVID-19 globally (122, 123). Older age, presence of a partner, and residence in a high-income country were thought to reduce the risk of sleep disturbances during the pandemic, whereas younger age, female sex, financial problems, and coexisting stress, anxiety, and depression enhanced the risk of sleep disturbances (124). To prevent further spread of the infection, governments of many countries, particularly Western countries, imposed social restrictions on the general population. The resultant changes in lifestyle associated with home confinement, such as the lack of morning sunlight exposure, lack of physical exercise, and excessive use of blue light devices at night, contributed to the changes in sleep behaviors of the general population (125). Of note, the effect of sleep hygiene-related behavior during the pandemic was larger in the young population than in the middle-aged or elderly population (126). Consistent with the studies by Wright et al. (127) and Marelliet et al. (128), we recently reported a significant delay in sleep phase, prolongation of total sleep time, and decrease in SJL from before to during the pandemic in 2222 Japanese participants from a young population cohort of 15–30 years of age (129). However, the worsening of insomnia and depression, as well as deterioration in health-related quality of life that was observed in Western countries was unexpectedly not observed in our study population. This could possibly be because of the smaller infection intensity and milder social restrictions in Japan than in Western countries at the survey point. However, considering that chronic sleep phase delay may result in subjective sleep problems and psychological distress (130) and that Japanese outpatients with DSWPD tended to show symptom aggravation because of a decrease in social zeitgeber during the pandemic (28), prolonged sleep phase delay along with extended social restrictions possibly impairs psychological distress and health-related quality of life. Fortunately, social restrictions owing to the COVID-19 pandemic have already ended in most countries. Nevertheless, we should carefully monitor sleep behaviors and their impact on the daytime function of the youth during the post-social restriction period. This is because the social advancement of the sleep phase and shortened nocturnal sleep time after the resumption of their work or school life may cause a significant psychological dysfunction (131).

### 4.3. Gaming disorder and DSWPD

Gaming is one of the most popular leisure activities. The COVID-19 pandemic greatly expanded the market of video game industry because people spent more time playing games worldwide (132, 133). Healthy gaming benefits education and training (134), however, some minority gamers experience negative consequences from excessive gaming (135). Gaming disorder (GD) is a relatively new mental disorder that shows the persistence of gaming behavior (online as well as offline) with a loss of control for gaming despite harm to individuals, and conflicts stemming from gaming and functional impairment (136).

In particular, online games allow players to select opponents and cooperating partners from all over the world, making it much easier for players to continue playing and enjoying games than offline games. In 2013, “internet gaming disorder (IGD)” was included in the “diagnostic and statistical manual of mental disorders, fifth edition (DSM-5)” (137) published by the American Psychiatric Association. In 2019, with strong encouragement from Japan, the World Health Organization (WHO) decided to adopt “Gaming Disorder (GD)” in the “international classification of diseases 11th Revision (ICD-11)” (136), and its use started in 2022. Although the content of the criteria differed, the concordance between GD and IGD diagnoses was reported to be fairly high (138).

As expected from elevated enthusiasm and prolonged exposure to blue light emitted from game devices at night, patients with GD have been reported to be frequently complicated with DSWPD (139). Adolescent patients with DSWPD need to be carefully checked to identify if there is a GD in their background. Interestingly, DSWPD and GD may have the following commonalities. First, both GD and DSWPD tend to appear in younger generations, including adolescents (140, 141). Second, not only patients with DSWPD but also those with GD are often complicated by neurodevelopmental disorders. Several studies have indicated that patients with GD are frequently complicated by either ADHD (142) or ASD (143). In such cases, treatment approach focusing on neurodevelopmental disorders should be included.

## 5. Current status and future challenges of DSWPD treatment

### 5.1. Chronobiological approaches

The 2007 CRSWD treatment guidelines of the AASM recommended light therapy as the first-line treatment for DSWPD (66). As mentioned earlier, exposure to morning light after achieving CBTmin advances the phase of the circadian rhythm. Generally, light therapy for patients with DSWPD is administered for 30 min to 2 h at 2500–10,000 lx during the time for phase advancement (or at 1–3 h before spontaneous awakening) (66). Expectedly, higher light intensity and longer duration of light exposure lead to a greater phase-shifting effect; however, this effect is nonlinear in humans (144, 145). Human circadian rhythms are most sensitive to short-wavelength blue light (~480 nm) (146, 147). Short-wavelength blue light is a more potent melatonin suppressor than long-wavelength light (148), and its application in light therapy has the potential to reduce light intensity and exposure time (149). However, effectiveness may also be lost if the light exposure timing is extremely late and out of the phase-advancing zone.

The side effects of light therapy on the skin and retina should also be noted. Although commercial light therapy products do not emit ultraviolet light, patients with eye diseases or those using photosensitizing drugs should be monitored regularly by ophthalmologists and dermatologists for underlying conditions during the administration of light therapy (150–152). Mania induction as a side effect of light therapy should also be considered (153). Melatonin release appears to decrease during depression and increase during mania (96, 154). Careful monitoring of psychiatric symptoms is crucial to maintain the safety of light therapy in patients with DSWPD and bipolar disorder (155). The AASM guidelines updated in 2015 noted that little evidence exists for the efficacy of light therapy in adults and recommended that light therapy should be administered only for DSWPD in children and adolescents after spontaneous awakening in combination with behavioral approaches provided by caregivers or others (156). As for the case of circadian-entrained DSWPD, no studies have investigated the effectiveness of light therapy in this phenotype.

The DSWPD treatment guidelines of AASM, which were revised in 2015 (156), recommend the use of melatonin and melatonin receptor agonists for the treatment of DSWPD in children, adolescents, and adults. Exogenous melatonin and its agonists have hypnotic effects along with decrease in CBT via the MT1 receptor and circadian phase resetting effect via the MT2 receptor. As for the role of the MT2 receptor, the PRC of melatonin administration on circadian rhythm is approximately 180° out of phase with that of light. Similar to CBTmin serving as an “inflection point” for the phase-delay and -advance effects of light, DLMO serves as an approximate inflection point for the phase-delay and -advance effects of melatonin (157). In patients with DSWPD, melatonin (0.3 mg) administration for a 4-week period between 1.5 and 6.5 h prior to DLMO showed phase advance of the circadian rhythm, wherein the magnitude of phase advance was strongly correlated with the time of melatonin administration and earlier administration times were more effective (158). Another study also showed that the administration of melatonin (5 mg) for 4 weeks between 19:00 and 21:00 h reduced sleep onset latency in patients with DSWPD (159). Evidence for the efficacy of melatonin in DSWPD, including the result of a meta-analysis (160) is being accumulated. Melatonin administration has been shown to improve comorbid depression and advance the melatonin secretory rhythm in patients with DSWPD (161). A small dose of melatonin administered 6–7 h before natural sleep onset (158, 162) or 5 h before DLMO (157) has been reported to be effective for the treatment of DSWPD. However, a consensus regarding the optimal timing, dose, and duration of melatonin administration has not yet been achieved.

Concerning the side effects of melatonin, the use of <10 mg/day in adults has been reported to be safe (163, 164). However, side effects, such as headache, somnolence, hypotension, hypertension, gastrointestinal upset, and worsening of alopecia areata, have been reported with high-dose usage (165). Cases of side effects, such as increased depressive symptoms (166) and decreased glucose tolerance (167) have also been reported. In the case of children, several studies have not found any adverse events with melatonin treatment in pediatric patients with DSWPD complicated by neurodevelopmental disorders (168–170). However, concerns exist about the effects of melatonin treatment on growth hormones during this developmental stage (171, 172) and the resulting potential adverse effects on reproductive function (173). One study (Meldos Trial) has reported no adverse events with reproductive development of the children when using melatonin at 0.3–10 mg doses (mean dose 2.69 mg) (174). In 2018, follow-up study of this trial also reported that adverse events were scarce but the study showed a tendency towards delayed puberty in the former and current users of melatonin (175). Another longitudinal study of melatonin treatment in 44 children with neurodevelopmental disorders showed that pubertal timing was considered within normal limits except in five children with severe neurodevelopmental disability, most of whom experienced precocious puberty prior to the start of melatonin treatment (170). A recent review of this area concluded that no consensus could be reached yet, as only a few studies with small samples have investigated the pubertal timing of melatonin users (176).

Ramelteon is the first melatonin receptor agonist developed as a hypnotic in Japan. Ramelteon has a high affinity for the MT1 receptor, which is considered to be involved in human sleep, and the MT2 receptor, which seems to regulate circadian rhythms (177, 178). Therefore, similar to melatonin, ramelteon is expected to exhibit therapeutic effects in DSWPD (179). Administration of 1–4 mg of ramelteon at 30 min before bedtime produces a phase advance. However, no difference in the effect was found between a dose of 8 mg and a placebo (180), suggesting that a small drug dose would be preferable for the treatment of DSWPD (181, 182). However, clinical evidence regarding the optimal timing of ramelteon administration for the treatment of DSWPD remains scarce. In addition to ramelteon, other melatonin receptor agonists are available. Tasimelteon is a melatonin receptor agonist that was approved as an orphan drug by the US FDA in 2010 for treating N24SWD in blind individuals. This drug exhibits high affinity for MT1 and MT2 melatonergic receptors in humans, which is similar to the action of melatonin or ramelteon (183, 184). Agomelatine (185) acts as both a melatonin receptor agonist and serotonergic receptor antagonist and was approved by the European Union for the treatment of depression in 2009. Agomelatine may promote sleep at night through its melatonergic effect and help maintain alertness during the day via its 5-HT_2C_ antagonistic effect (184). However, little evidence exists on agomelatonine’s ability to improve circadian rhythms when compared to other melatonergic drugs.

Among other DSWPD treatment methods, chronotherapy (60), in which sleep is intentionally delayed for 3 h each day to fix the sleep–wake rhythm to the desired time, has been formerly advocated. Although the literature on chronotherapy is scarce, few studies have reported cases that were effectively treated with chronotherapy. One case report showed that chronotherapy improved nighttime sleep and daytime psychiatric symptoms in children with attention deficit disorder complicated by DSWPD (186), while another case report found that the combination of chronotherapy and light therapy was effective in the treatment of DSWPD (187). However, chronotherapy is labor-intensive and carries the risk of developing N24SWD (188). Therefore, this therapy is not currently recommended in the AASM guidelines.

### 5.2. Newer candidates for DSWPD treatment

Aripiprazole is an antipsychotic drug that acts as a partial agonist of D2 receptors (189), but it appears to have no direct chronobiological action. However, a low dose of aripiprazole (3 mg or less) was reported to be effective in enabling patients with DSWPD to wake up in the morning (190). Although the detailed mechanism of its action is unknown, this drug appears to help in waking up at the desired time in the morning, which leads to a decrease in sleep time and consequent advancement of the sleep phase (191). Furthermore, aripiprazole is an effective adjunctive therapy for major depressive disorders (192). As previously mentioned, patients with DSWPD often have complications, such as depressive symptoms or prolonged sleep duration. Therefore, aripiprazole may be a new potential treatment option for DSWPD. Aripiprazole has fewer side effects than other antipsychotics and is increasingly prescribed to children, but drowsiness, extrapyramidal effects, metabolic effects, and weight gain should be noted (193). Although this drug is only used at low doses in DSWPD, it is an off-label prescription and requires careful monitoring of side effects in children and adolescents.

To date, the treatment of DSWPD has mainly focused on the chronobiological background. However, DSWPD is often recurrent and likely to follow a chronic course (19, 66), and either environmental or psychosocial factors may also contribute to the development and perpetuation of the disorder. Particularly in adolescent DSWPD, late work schedule, involvement in extracurricular activities, exposure to indoor lighting during evening hours (194), and/or delay in weekend wake-up time (195) may affect treatment responses (196). In these situations, a carefully individualized approach to change problematic situations is necessary. Furthermore, repeated exposure to frustration about sleep initiation can lead to psychological hyperarousal at night, which may contribute to the perpetuation of the disorder. Considering this process and that patients with DSWPD are likely to have elevated neuroticism (83), a cognitive-behavioral approach consisting of stimulus control, sleep hygiene education, cognitive restructuring, and mindfulness-based stress reduction to address sleep latency, in conjunction with the chronobiological approach, may become a treatment option for DSWPD. However, evidence for the effectiveness of combination treatment confirmed through randomized controlled trials on a large number of cases remains scarce. Of note, cognitive and behavioral approaches are also possible candidates for the treatment of circadian entrained-DSWPD. Given that, the likelihood of a favorable response to chronobiological treatment is quite low in patients with a lack of social zeitgebers, such as school attendance and employment or those without motivation for treatment; thus, less complex interventions should be considered for patients with these characteristics (156). In addition, ensuring diversity in social institutions so as to provide accommodation for the circadian preference of patients with DSWPD may be an important choice for some refractory cases (197).

## 6. Conclusion

From a psychiatric perspective, we reviewed the sleep behavior of adolescents and young adults, the psycho-behavioral characteristics of DSWPD in this young generation, and the association of DSWPD with psychiatric disorders. The pathogenesis of DSWPD is heterogeneous, with many mechanisms yet to be elucidated. The phenotype of DSWPD (including the presence or absence of circadian entrainment and phase angle expansion) varies depending on the interrelationship among various factors, including biological, social, and environmental factors, psycho-behavioral characteristics, and psychiatric disorders. DSWPD is a recurrent disorder, and its treatment is labor-intensive and time-consuming. Conventional DSWPD treatment has focused on biological factors; however, individually optimized treatment that considers not only the chronobiological factors but also psychological factors as well as the lifestyle and environment of young people should be developed.

## Author contributions

All authors listed have made a substantial, direct, and intellectual contribution to the work and approved it for publication.

## Funding

This research was funded by a grant from the Japan Society for the Promotion of Science (KAKENHI) (grant number: JP21K13703).

## Conflict of interest

KF reported personal fees from Eisai., Ltd. and MSD outside the submitted work. YT reported lecture fees from Takeda Pharmaceutical, Sumitomo Pharma, Otsuka Pharmaceutical, Meiji Seika Pharma, Kyowa Pharmaceutical, Eisai, MSD, and Yoshitomi Pharmaceutical outside the submitted work. KM reported personal fees from Eisai, Meiji Seika Pharma, MSD, Otsuka Pharmaceutical, Takeda Pharmaceutical, and Yoshitomi Pharmaceutical outside the submitted work. AS reported personal fees from Eisai and Sumitomo Pharma outside the submitted work. IO reported grants from NEC Solution Innovators Co., Ltd. and Infocom Co.; personal fees from Otsuka Pharmaceutical MSD, and Eisai.; and consultation fees from NEC Solution Innovators Co., Ltd. and Suntory Wellness Ltd. outside the submitted work. YK reported lecture fees from Eisai outside the submitted work. YI reported personal fees from Eisai, Otsuka Pharmaceutical, Takeda Pharmaceutical, Astellas Pharma Inc., and MED K.K. and grants from Philips Japan Co., Ltd., Koike Medical Co., Ltd., and Teijin Pharma Ltd. outside the submitted work.

The remaining author declares that the research was conducted in the absence of any commercial or financial relationships that could be construed as a potential conflict of interest.

## Publisher’s note

All claims expressed in this article are solely those of the authors and do not necessarily represent those of their affiliated organizations, or those of the publisher, the editors and the reviewers. Any product that may be evaluated in this article, or claim that may be made by its manufacturer, is not guaranteed or endorsed by the publisher.
